# Dynamics of acylcarnitines, hypoglycin A, méthylènecyclopropylglycine and their metabolites in a Kladruber stallion with atypical myopathy

**DOI:** 10.1080/01652176.2022.2126537

**Published:** 2022-09-23

**Authors:** Petr Jahn, Dana Dobešová, Radana Brumarová, Katarína Tóthová, Andrea Kopecká, David Friedecký

**Affiliations:** a Equine Clinic, Faculty of Veterinary Medicine, University of Veterinary Sciences, Brno, Czech Republic; b Laboratory for Inherited Metabolic Disorders, Department of Clinical Biochemistry, University Hospital Olomouc and Faculty of Medicine and Dentistry, Palacký University Olomouc, Olomouc, Czech Republic

**Keywords:** Equine atypical myopathy, multiple acyl-CoA dehydrogenases deficiency, acylcarnitines, hypoglycin A, méthylènecyclopropylglycine, Acer pseudoplatanus, dry blood spot, mass spectrometry

## Abstract

Equine atypical myopathy (AM also referred to as multiple acyl-CoA dehydrogenases deficiency [MADD]) is thought to be caused by toxins metabolized from hypoglycin A (HGA) and méthylènecyclopropylglycine (MCPrG). HGA is contained in the seeds and seedlings of the sycamore tree (*Acer pseudoplatanus*); MCPrG has so far only been confirmed in seeds. Among other things, these substances can disrupt the fatty acids β-oxidation pathway with the subsequent accumulation of certain acylcarnitines. The tentative diagnosis is based on anamnesis and clinical signs and can be verified by the detection of elevated creatine kinase activity, specific profile of acylcarnitines and the presence of HGA, MCPrG conjugates and/or their metabolites in peripheral blood and/or urine. Dry blood spots were collected for 15 days from a 3.5-year-old stallion which had been affected by AM and, as a control group, from twelve healthy horses. Two mass spectrometry methods were used for the analysis of 31 acylcarnitines, carnitine, HGA, MCPrG and their metabolites. HGA and six increased acylcarnitines were detected in the patient’s blood throughout the monitoring period. Nine acylcarnitines were strongly correlated with HGA. Multivariate statistical analysis showed a clear separation of samples from the AM horse, where the metabolic profile tended to normalization in the later days after intoxication. Due to the longer persistence in the blood, the detection of HGA and elevated acylcarnitines profile appear to be an appropriate tool to confirm the diagnosis of AM, compared to metabolic products of HGA and MCPrG even in advanced cases.

## 1. Introduction

Equine atypical myopathy (AM) and seasonal pasture myopathy (SPM) (also referred to as multiple acyl-CoA dehydrogenases deficiency [MADD]) are rhabdomyolyses of grazing horses that occur mainly in autumn and the following spring. Clinical signs include colic, muscular weakness, stiffness, trembling, sweating, myoglobinuria, and recumbency (Finno et al. 2006; van Galen, Marcillaud Pitel, et al. 2012). The causative agents of AM and SPM are thought to be toxins contained in seeds and seedlings of the sycamore tree (*Acer pseudoplatanus*) (Votion et al. 2014) and the box elder tree (*Acer negundo*) (Valberg et al. 2013). Hypoglycin A (HGA) as one of the toxins is, after ingestion, transformed into a toxic metabolite methylenecyclopropylacetyl-CoA (MCPA-CoA) which is known to cause deficiency of multiple acyl-CoA dehydrogenases leading to the disruption of the first step of β-oxidation in fatty acids metabolism (Westermann et al. 2008; Sponseller et al. 2012) and leucine degradation pathway (Luís et al. 2011). Another substance, méthylènecyclopropylglycine (MCPrG), is metabolically transformed to methylenecyclopropylformyl-CoA (MCPF-CoA), which causes the inhibition of the second step of β-oxidation (Bochnia et al. 2019). Consequently, certain fatty acyls conjugated with carnitine and glycine accumulate in the blood and urine of the affected horses (Westermann et al. 2008; Valberg et al. 2013; Votion et al. 2014).

Diagnosis of AM is based on the history and clinical signs, the detection of elevated creatine kinase (CK) activity confirming rhabdomyolysis and the presence of HGA and MCPA conjugates in peripheral blood (Valberg et al. 2013; Votion et al. 2014; Bochnia et al. 2015; Sander et al. 2016). Since analyses of HGA and MCPA conjugates are expensive and not commonly available to equine practitioners as part of a routine laboratory examination, Sander et al. (Sander et al. 2018) proposed the use of the quantification of certain elevated serum acylcarnitines by using a dry blood spot (DBS) for a rapid diagnosis of AM. This test is performed routinely in laboratories that screen human neonates for inborn metabolic errors.

The information on the dynamics of HGA, MCPrG and conjugates of their metabolites and changes in acylcarnitine concentrations in the peripheral blood of horses suffering from AM during the progression of the disease is limited. Bochnia et al. (Bochnia et al. 2018) described changes in HGA and MCPA-carnitine in the peripheral blood of a horse suffering from AM for three days of the disease progression. A study examining changes in the acylcarnitines profile in two horses with AM on days 7 and 9 from the diagnosis was recently published by Mathis et al. (Mathis et al. 2021). Of note, some of these metabolites have been observed in one person after ingestion of canned ackee (*Blighia sapida*) (Sander et al. 2020). This fruit, popular in some tropical countries, also contains HGA and MCPrG.

The aim of the current study was to monitor CK activity and the concentrations of HGA, MCPrG, MCPA-carnitine, MCPA-glycine, MCPF-carnitine, MCPF-glycine, carnitine, and 31 acylcarnitines in the peripheral blood of a horse affected by AM over a period of 15 days. The concentrations of the acylcarnitines in peripheral blood were compared with twelve healthy horses on pastures without access to sycamore or box elder trees.

## 2. Case description

A Kladruber black stallion aged 3.5 years (body weight 535 kg) was referred to our clinic by a veterinary practitioner on 1st October 2018. According to the history, the horse was found on the pasture surrounded by several sycamore trees in the morning on the day of admission standing, lethargic, anorectic, and reluctant to move. The previous day, the horse was without clinical signs of any disease.

After admission to the clinic, the horse showed stiff movement and weakness. Body temperature was 36.6 °C, heart rate 51 beats per minute and respiratory rate 12 breaths per minute. Mucous membranes were pink, capillary refill time 2 s, auscultation of the thorax and peristalsis was normal. Ultrasonography of the abdomen and thorax was without pathology. Rectal examination revealed a distended urinary bladder; other organs of the abdominal cavity were without pathology. Blood samples were collected for haematology and biochemistry. Approximately 5 L of dark brown urine was obtained by catheterisation of the urinary bladder (examination by reagent strip: pH 5, glucose 55 mmol/L, haemoglobin/myoglobin +++). Haematology revealed neutrophilia (6.9 G/L) with elevated bands (0.82 G/L); other haematological parameters were within the laboratory’s reference ranges. Biochemistry revealed hyperglycaemia (15.3 mmol/L), elevated concentrations of lactate (2.8 mmol/L), total bilirubin (49.3 µmol/L), and increased activity of muscle enzymes: creatine kinase (CK, 516,879 IU/L), lactate dehydrogenase (LDH, 15,545 IU/L), and aspartate aminotransferase (AST, 11,492 IU/L). Plasma concentrations of sodium (131 mmol/L) and ionized calcium (1.05 mmol/L) were decreased. Plasma concentrations of total protein, albumin, triacylglycerols, urea, creatinine, chloride ions, and the activity of alkaline phosphatase and γ-glutamyltransferase were within the reference limits. Partially compensated metabolic acidosis was detected: the pH of venous blood was 7.263, the concentration of bicarbonate 15.5 mmol/L, base excess −11.0 mmol/L, pCO2 4.48 kPa. The activity of glutathione peroxidase was within the reference range (11,958 IU/L).

Based on the history (presence of sycamore trees near the pasture and the appearance of atypical myopathy on this farm in previous years), clinical signs, pigmenturia and very high CK activity, a diagnosis of atypical myopathy was suspected.

An intravenous cannula was inserted into the jugular vein and the horse was placed in a box at the clinic. Acid-base status was corrected by intravenous administration of fluids (normal saline solution and sodium bicarbonate), and the values of other biochemical parameters (except the activity of muscle enzymes) returned to the reference values within 24 hours. Intravenous fluids (40 L/day) were applied for the following eight days. Elevated body temperature (38.5 − 38.9 °C) was recorded from day 3 to 7 and transient leukopenia with left shift (3.8 G/L; bands 0.34 G/L) on day 3; therefore, sulfadiazine with trimethoprim (30 mg/kg BW iv BID) was administered for 10 days. Other treatments consisted of the administration of flunixin (1.1 mg/kg BW iv SID for day 1–2), ketoprofen (2.2 mg/kg BW iv SID for days 2–7) and morphine (0.05 mg/kg BW im TID for days 2–7). Selenium (0.5 mg/kg BW po) and vitamin E (0.5 mg/kg BW po) were applied for the first three days, multi-B vitamin (Kombisol B komplex, Trouw Nutrition Biofaktory, Praha, Czech Republic, 20 ml po) for the first five days, and carnitine (8,800 mg po) for the first ten days.

Subcutaneous oedema of the head developed in the horse on the second day of hospitalization due to its low carriage. When the head was supported in a higher position, the oedema disappeared. The horse was able to eat from the day of admission, but chewing hay was slow. Feed intake improved after three days of hospitalization. On the third day, the urine colour changed to normal, and after five days of hospitalisation, the horse showed a tendency to move spontaneously in the box and increased its feed intake. On the fifteenth day of hospitalisation, the horse walked for 5 minutes. The clinical condition gradually improved, and the horse was discharged from the clinic after 23 days. Three years after hospitalisation, the horse had no health problems and was used for riding.

Samples of the jugular blood were collected repeatedly during hospitalisation for routine biochemistry and haematology. Part of each sample was used for the analysis of HGA, MCPrG, MCPA-carnitine, MCPA-glycine, MCPF-carnitine, MCPF-glycine and concentrations of various acylcarnitines.

## 3. Control animals

Twelve healthy horses grazing on pastures without sycamore and/or box elder trees growing on or near the pasture (8 mares, 4 geldings; 5 Haflingers, 1 Paint Horse, 1 Czech Warmblood, 1 Dutch Warmblood, 1 Irish Cob, 1 Draft Horse, 1 Appaloosa x Thoroughbred, and 1 Hucul) were used as controls to compare blood acylcarnitines concentrations.

## 4. Sample preparation and laboratory diagnosis

Haematological and biochemical routine diagnostics were performed in the Small Animal Clinical Laboratory of the University of Veterinary and Pharmaceutical Sciences Brno. Complete haematological and biochemical panels were examined only on the day of admission and on five following days. CK activity was determined on days 1 − 15 to assess the progression of rhabdomyolysis. A drop of blood from each sample was spotted on a filter paper card and allowed to dry at room temperature. With the informed consent of the horse owner, these DBS were used for the analysis of HGA, MCPrG, MCPA-carnitine, MCPA-glycine, MCPF-carnitine, MCPF-glycine and acylcarnitines. The same procedure was used to prepare single DBS in the 12 healthy control horses with the informed consent of their owners. The samples were prepared as described elsewhere (Sander et al. 2016; Karlíková et al. 2018). The analysis of 31 acylcarnitines and carnitine in the affected horse and healthy controls was performed using a flow injection analysis mass spectrometry (FIA-MS). The full names of acylcarnitines are shown in Supplementary Table S1. HGA, MCPrG and MCPA/MCPF conjugates were measured in the affected horse by high-performance liquid chromatography coupled with tandem mass spectrometry (LC-MS/MS). Detailed information about both the analytical methods used is described in the paper of Karlíková et al. (Karlíková et al. 2018). The MS/MS transitions (mass to charge ratio of precursor and product ions) for the butyl esters of toxins were as follows: HGA (198/74); MCPrG (184/111), MCPA-carnitine (312/85); MCPA-glycine (226/74), MCPF-glycine (212/81) and MCPF-carnitine (298/85) (Bochnia et al. 2019). The form of acylcarnitine nomenclature used (e.g. C5-DC + C6-OH) means that both analytes were detected in the same peak due to the same MS/MS transitions.

**Table 1.** Plasma CK activity and blood concentrations of HGA, MCPA-carnitine, MCPA-glycine, and MCPF-carnitine in AM horse (reference range of CK is 110–250 IU/L).

Day of hospitalisation	CK activity [IU/L]	Concentration of the following analytes [µmol/L]:			
		HGA	MCPA-carnitine	MCPA-glycine	MCPF-carnitine
1	516,879	6.574	0.083	0.565	0.208
2	804,101	2.335	0.022	0.287	0.074
3	737,568	1.173	0.008	0.153	0.035
4	396,276	0.557	0.006	<dl	0.032
5	145,665	0.224	<dl	<dl	0.005
6	27,534	0.230	<dl	<dl	<dl
7	10,710	0.220	<dl	<dl	<dl
8	5,821	0.219	<dl	<dl	<dl
9	3,409	0.150	<dl	<dl	<dl
10	2,244	0.131	<dl	<dl	<dl
12	1,185	0.076	<dl	<dl	<dl
15	586	0.091	<dl	<dl	<dl

CK, creatine kinase; HGA, hypoglycin A; MCPA-carnitine, methylencyclopropylacetyl-carnitine; MCPA-glycine, methylencyclopropylacetyl-glycine; MCPF-carnitine, methylencyclopropylformyl-carnitine; dl, detection limit.

## 5. Data treatment and statistical analysis

Data were statistically evaluated in the R program (version 4.0.3, www.R-project.org) using the Metabol package (AlzbetaG) and in the SIMCA software (version 15.0, Umetrics, Umeå, Sweden). Natural logarithmic transformation and mean centering were used for multivariate statistical analysis (principal component analysis – PCA). Shapiro-Wilk normality test followed by non-parametric Spearman correlation analysis were performed on the raw data by GraphPad Prism software (version 9.0). The same normality test was also applied to the control horse data. The results are summarized in Supplementary Table S1. For visualization of changes in analytes concentration and enzyme activity over time, MS Excel program (Microsoft Office 2019) was used.

## 6. Results

**Figure 1.**
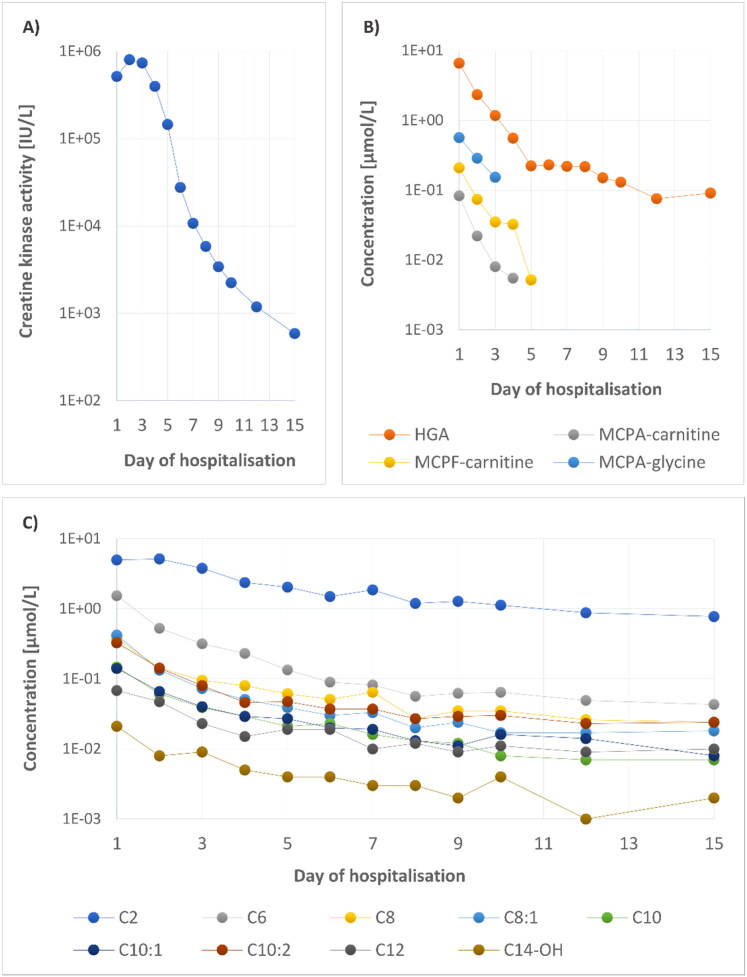
(A) Plasma CK activity in the affected horse during hospitalization. (B) HGA, MCPA-carnitine, MCPA-glycine, and MCPF-carnitine concentrations over time. (C) Concentration changes of nine acylcarnitines strongly correlating with dynamic of HGA.

The increased CK activity in the affected horse persisted for 15 days (Table 1, Figure 1A), with a peak on the second day of hospitalisation. The dynamic of blood concentrations of HGA, MCPA-carnitine, MCPA-glycine, and MCPF-carnitine during the disease is documented in Table 1 and Figure 1B. MCPrG and MCPF-glycine were not detected. Detection limits were calculated as: signal-to-noise ratio = 3, for HGA, MCPA, MCPA-carnitine, MCPA-glycine, and MCPF-carnitine: 60, 5, 6, 9, 4 nmol/L, respectively.

**Figure 2.**
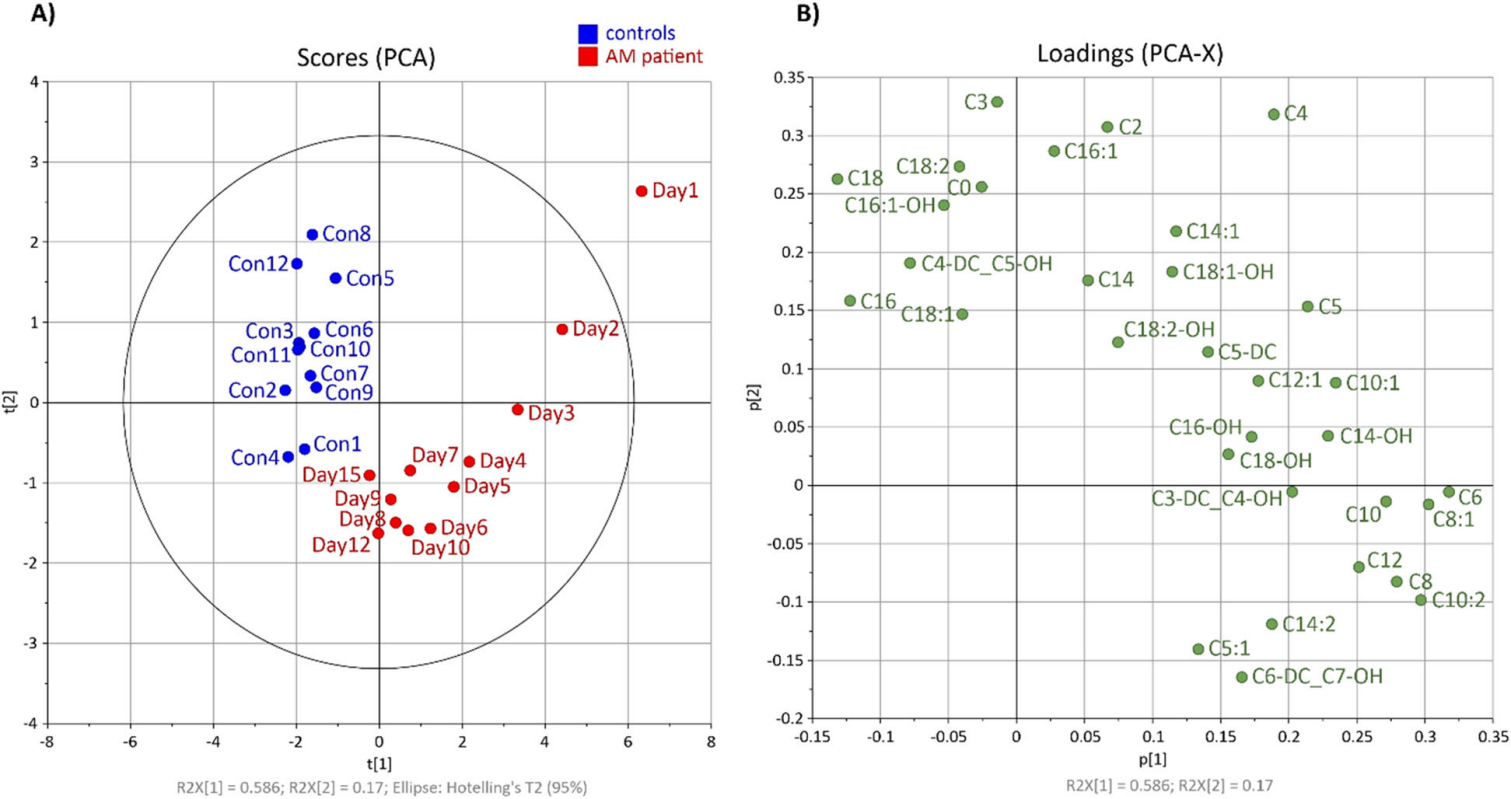
Principal component analysis: (A) score plot of AM-affected horse during 15 days of hospitalisation (red) and 12 samples from healthy controls (blue); (B) loading plot of carnitine and 31 acylcarnitines.

Unsupervised principal component analysis (Figure 2A and B; score and loading plots) constructed from the acylcarnitine dataset shows a clear separation between multiple samplings obtained from the affected horse and healthy controls (cumulative explained variation is 69%). As expected, the profile of acylcarnitines was most altered in the first blood sample (Day 1) from the affected horse and gradually normalized in the subsequent samples (Day 2–15) (Figure 2A). Alterations in concentrations of carnitine and 31 acylcarnitines over time and their comparison with 95th percentile of the 12 healthy controls are shown in Table 2 and Supplementary Table S1. The loading plot shows the most discriminating medium-chain acylcarnitines which were increased in AM-affected horse (Figure 2B). The elevation of C6, C6-DC + C7-OH, C8:1, C10:2, C12 and C14:2 concentrations persisted until day 15 (Supplementary Table S1).

**Table 2.** Blood concentrations of carnitine and 31 acylcarnitines in the AM-affected horse on the first day of hospitalization and 95^th^ percentile of healthy controls [µmol/l] with elevated values in bold.

Acylcarnitine	Full name	AM patient	Healthy controls
C0	L-carnitine	9.535	13.120
C2	acetyl-L-carnitine	**4.994**	4.410
C3	propionyl-L-carnitine	**0.688**	0.593
C3-DC + C4-OH	malonyl-L-carnitine/hydroxybutyryl-L-carnitine	**0.175**	0.051
C4	butyryl-L-carnitine	**6.701**	0.596
C4-DC + C5-OH	succinyl-L-carnitine/hydroxyvaleryl-L-carnitine	0.128	0.291
C5	valeryl-L-carnitine	**9.625**	1.000
C5:1	tiglyl-L-carnitine	**0.034**	0.031
C5-DC	glutaryl-L-carnitine	**0.719**	0.301
C6	hexanoyl-L-carnitine	**1.537**	0.023
C6-DC + C7-OH	adipoyl-L-canitine/hydroxyheptanoyl-L-carnitine	**0.069**	0.034
C8	octanoyl-L-carnitine	**0.377**	0.024
C8:1	octenoyl-L-carnitine	**0.421**	0.010
C10	decanoyl-L-carnitine	**0.147**	0.008
C10:1	decenoyl-L-carnitine	**0.141**	0.013
C10:2	decadienoyl-L-carnitin	**0.327**	0.012
C12	lauroyl-L-carnitine	**0.068**	0.007
C12:1	dodecenoyl-L-carnitine	**0.057**	0.013
C14	tetradecanoyl-L-carnitine	**0.077**	0.056
C14:1	tetradecenoyl-L-carnitine	**0.073**	0.019
C14:2	tetradecadienoyl-L-carnitine	**0.021**	0.006
C14-OH	hydroxytetradecaenoyl-L-carnitine	**0.021**	0.004
C16	palmitoyl-L-carnitine	0.502	1.048
C16:1	hexadecenoyl-L-carnitine	**0.054**	0.047
C16-OH	hydroxyhexadecanoyl-L-carnitine	**0.033**	0.008
C16:1-OH	hydroxyhexadecenoyl-L-carnitine	0.041	0.057
C18	stearoyl-L-carnitine	0.411	0.843
C18:1	oleoyl-L-carnitine	0.401	0.759
C18:2	linoleoyl-L-carnitine	0.045	0.093
C18-OH	hydroxyoctadecanoyl-L-carnitine	**0.010**	0.005
C18:1-OH	hydroxyoctadecenoyl-L-carnitine	**0.030**	0.014
C18:2-OH	hydroxyoctadecadienoyl-L-carnitine	**0.017**	0.016

Correlation analysis of the acylcarnitines and the primary toxin HGA showed a strong correlation (r > 0.90) for nine acylcarnitines with an even carbon chain (C2, C6, C8, C8:1, C10, C10:1, C10:2, C12, C14-OH; Figure 1C, Supplementary Table S1).

## 7. Discussion

Atypical myopathy is a disease with a mortality rate of around 75% or higher (Votion et al. 2007; Van Galen et al. 2010; van Galen, Marcillaud Pitel, et al. 2012). The clinical and laboratory signs of the horse described in our study were similar to those reported in other studies: stiffness, reluctance to move, pigmenturia and elevation of CK activity. Increased or persistent recumbency, which is reported as a predictor of poor prognosis (van Galen, Saegerman, et al. 2012; Boemer et al. 2017; González-Medina et al. 2017), was not observed in our patient.

Enzyme activity of LDH, AST and especially CK in horses with AM is usually dramatically increased (Votion et al. 2007; Votion and Serteyn 2008; Westermann et al. 2008; Valberg et al. 2013; Votion et al. 2014). Plasma CK activity is a specific indicator of rhabdomyolysis with a peak at 6 − 12 hours after muscle injury and a half-life of approximately 12 hours (Toutain et al. 1995). In our case the continued increase on the second day of hospitalisation suggests ongoing muscle damage. A similar increase in CK activity after the beginning of treatment was also observed in two other surviving horses in the study of Karlíková et al. (Karlíková et al. 2016). From the fourth day of hospitalization onwards, the CK activity decreased to approximately half the value of the previous day. Its dynamic was probably influenced by the infusion therapy that was applied to the horse during the first eight days of hospitalization.

Other differences in the biochemical profile of our case on the day of admission were similar to those previously reported. Elevated blood glucose and lactate concentrations have been observed in most cases (Votion et al. 2007; Westermann et al. 2008) and are considered predictors of a poor prognosis (Dunkel et al. 2020). The reason for hyperglycaemia is believed to be a high ability to mobilize glucose from hepatic glycogen stores and may also reflect insulin resistance (Votion et al. 2007; Sponseller et al. 2012).

Since 2013, in addition to the clinical signs listed above, elevated CK activity and concentrations of various acylcarnitines, the detection of HGA or its metabolites in the blood of affected horses has been added to the gold standard for the diagnosis of AM (Valberg et al. 2013; Votion et al. 2014; Bochnia et al. 2015; Sander et al. 2016). Information on how long HGA, MCPrG and their metabolites can be detected in the blood of horses after ingestion of sycamore seeds or seedlings is very sparse. Gröndahl et al. (Gröndahl et al. 2015) reported the detection of HGA in horses one month after their relocation to a different pasture, but it is not clear whether this pasture was without sycamore trees and there is no information on the concentrations of HGA metabolites in these horses. In 2018, Bochnia and colleagues observed a rapid decrease in HGA and an increase in MCPA-carnitine concentrations in the serum of an affected horse, but these changes were only monitored for two days of the disease before the horse was euthanized (Bochnia et al. 2018). Among other things, the time course, and metabolic effects of HGA, MCPrG, several organic acids and acylcarnitines (short and medium-chain) were studied in urine and serum samples from one human volunteer after consuming canned ackee (*Blighia sapida*) (Sander et al. 2020). In our study, HGA was detected in the blood of the affected horse for a longer time than its metabolites – to 15th day of hospitalization, followed by MCPA-carnitine and MCPA-glycine (these were detected until day 4 and 3, respectively; Table 1, Figure 1B). However, the measurement is limited by the chosen analysis and the sample preparation method used. The concentration of toxins and metabolites in the blood of our patient can also be affected by treatment, especially infusion therapy and carnitine supplementation.

The published values of serum HGA concentrations in AM cases range from 0.23 µmol/L (Carlier et al. 2015) to 60 µmol/L (Bochnia et al. 2015). The concentration of HGA in our horse on the day of admission to the clinic was within this range (Table 1). In both mentioned studies, the timing of blood sampling in relation to the onset of clinical symptoms has not been given. Furthermore, analyses were performed in various laboratories using different materials (blood, serum, or plasma), so it is not possible to compare these values accurately. MCPA-carnitine concentrations in published studies were lower and more variable than HGA concentrations and varied from 0.95 nmol/L (Votion et al. 2014) to 1.18 µmol/L (Sander et al. 2016). In our case-report, MCPA-carnitine was detected up to day 4 and its concentrations were in the range of those published previously. Concentrations of the second MCPA conjugate (MCPA-glycine), were higher on the same day than for MCPA-carnitine (Table 1), similar to the study by Bochnia et al. (Bochnia et al. 2019) describing the metabolites of HGA and MCPrG in 14 AM-affected horses. In our study, the maximum concentration of MCPA-glycine (0.565 µmol/L on day 1), was in the range (0.170 − 4.649 µmol/L) that was published by Bochnia et al. (Bochnia et al. 2019).

In AM research, less attention has been paid to MCPrG conjugates in the blood of AM-affected horses. MCPrG was not identified in our patient, but its metabolite MCPF-carnitine was detected up to day 5. In our patient, the concentrations of MCPF-carnitine (Table 1, Figure 1B), (maximum 0.208 µmol/L on the first day) were below the values from the study of Bochnia et al. (Bochnia et al. 2019), who detected MCPF-carnitine in the blood samples of all 14 AM-affected horses (range 0.990 − 18.903 µmol/L). MCPrG concentrations in serum of affected horses were extremely variable and always lower than HGA levels in the study by Bochnia et al. (Bochnia et al. 2019). Interestingly, the maximal value of MCPF-carnitine was found in the horse which had only a trace concentration of MCPrG isomer A in this study. A possible explanation for the low (undetectable) MCPrG content in body fluids could be the rapid and complete transformation to its metabolites after absorption in the gastrointestinal tract (Bochnia et al. 2019).

MCPF-glycine was also detected in AM-affected horses (Bochnia et al. 2019). However, it was not the major MCPrG-metabolite found; rather, MCPF-carnitine was measured in concentrations that exceeded those of MCPF-glycine by one to two powers of ten. In our study, MCPrG and its metabolite MCPF-glycine were not detected. The lower sensitivity of our methods compared to the above methods is probably due to the use of DBS. During sample preparation, 3 µL of blood (for DBS) are taken for analysis and therefore the sample is an order of magnitude more diluted than in the methods using serum or plasma.

The profile of acylcarnitines (Figure 2A and B) was measured over 15 days after the first signs of HGA/MCPrG poisoning. In most studies describing hypoglycin A intoxication, an increase in short (C2–C5), medium (C6–C13) and long-chain (C14–C20) acylcarnitines was observed in affected horses (Westermann et al. 2008; Valberg et al. 2013; Votion et al. 2014; Karlíková et al. 2016; Sander et al. 2016, 2018; Mathis et al. 2021). However, in 2013 Valberg et al. observed elevated C5-C10 and a slight increase of C12 and C16 in one surviving AM patient (Valberg et al. 2013). In the study of Votion et al. (2014) mild increases of C4, C5, C6 and C8 above the reference range were present in the horse with the lowest MCPA-carnitine concentration. The occurrence of acylcarnitines in the time profile was recently described in the study of Mathis et al. (Mathis et al. 2021), who focused on the levels of these metabolites in two horses with AM. In one of them, an increased C8 was detected by the 4th day after diagnosis. The second horse showed elevated levels of six analytes (C3-DC + C4-OH, C5, C6, C8, C8:1, C10) until Day 7. In our case-report (Figure 1C), the rate of HGA degradation correlated most strongly (r > 0.90) with the reduction of nine acylcarnitines (C2, C6, C8, C8:1, C10, C10:1, C10:2, C12, C14-OH). For the other acylcarnitines, the Spearman correlation coefficient was calculated as less than or equal to 0.80.

Treatment of AM is not specific and aims to restore the circulating volume, correct acid-base and electrolyte imbalances, provide energy to the affected muscles, eliminate toxin and toxic metabolites and, depending on the patient’s condition, alleviate pain and discomfort and prevent further injuries (Fabius and Westermann 2018; Witkowska-Pilaszewicz et al. 2019). Therefore, treatment of our patient consisted mainly of fluids, NSAIDs, morphine, multi vitamin B, vitamin E and selenium administration. In addition, it is recommended to administer carnitine, which promotes β-oxidation of long-chain fatty acids in the mitochondria. The elimination of increased acyl residues is supported by activating their transfer from the cytoplasm to the mitochondria, by influencing the CoA-SH/Acyl-CoA ratio and also by increasing the plasma concentration of leptin (Fabius and Westermann 2018). In our patient, the cause of the fever and leucocytosis was not found, but the aspiration of oral contents into the lower respiratory tract was suspected. Sulfadiazine with trimethoprim probably solved this problem.

In summary, in this study, we were able to determine concentrations of carnitine and 31 acylcarnitines in the DBS of a horse suffering from AM compared to twelve healthy horses. Six of them had long-elevated persistence in peripheral blood of especially C6, C6-DC + C7-OH, C8:1, C10:2, C12 and C14:2. Furthermore, concentrations of nine acylcarnitines with an even carbon chain showed a strong correlation with HGA dynamics. In addition, like acylcarnitines, the blood HGA concentration has been observed to be increased for a long time compared to its metabolites. The detection of HGA and assessing the profile of acylcarnitines appears to be a suitable option to confirm the diagnosis of AM, as determined by clinical trials and possible medical history. The use of DBS as biological material has proven useful due to the easy availability and speed of subsequent testing of profile of acylcarnitines and the presence of HGA in the blood. The current study is based on close monitoring of one equine patient with AM, and to confirm these results, it would be useful to include more subjects in future studies.

## Supplementary Material

Supplemental Material

## Data Availability

The attachment presented in this study is included in the Supplementary material. Other questions can be directed to the corresponding authors.
